# Orthognathic Surgery in Patients With Von Willebrand’s Disease: A Report of Four Cases and Literature Review

**DOI:** 10.7759/cureus.57305

**Published:** 2024-03-31

**Authors:** Marika Sato, Hayato Hamada, On Hasegawa, Yoko Kawase-Koga, Daichi Chikazu

**Affiliations:** 1 Department of Oral and Maxillofacial Surgery, Tokyo Medical University, Tokyo, JPN; 2 Department of Oral and Maxillofacial Surgery, Tokyo Women's Medical University, Tokyo, JPN

**Keywords:** occlusion, bilateral sagittal split osteotomy, bleeding disorder, von willebrand’s disease, orthognathic surgery

## Abstract

Von Willebrand’s disease (VWD), characterized by quantitatively or qualitatively abnormal von Willebrand factor (VWF), is the most common inherited bleeding disorder. There is limited evidence of treatment using orthognathic surgery in patients with VWD. This report focuses on four patients with VWD who underwent orthognathic surgery and received Factor VIII/VWF concentrates (Confact F) preoperatively. One patient with type 3 (severe) VWD underwent delayed extubation owing to laryngeal edema and exhibited epistaxis thereafter. No perioperative complications were observed in any of the other patients. Two of the four patients were diagnosed with VWD during preoperative screening. Most young adults do not experience general anesthesia and, therefore, may not have undergone blood tests at a hospital. Thus, preoperative screening and adoption of a multidisciplinary approach to orthognathic surgery is important in patients with bleeding disorders such as VWD. Close communication between anesthetists, surgeons, and hematologists is essential to ensure effective management during the perioperative period.

## Introduction

Von Willebrand’s disease (VWD), a common inherited bleeding disorder characterized by quantitative or qualitative deficiencies in the von Willebrand factor (VWF), has a prevalence rate of approximately 1% in the general population [1]. However, only 0.01% of these patients are symptomatic [2]. VWD can be classified into types 1 (i.e., mild; characterized by partial quantitative VWF deficiency); type 2 (moderately severe; characterized by qualitative VWF deficiency), and type 3 (severe; characterized by total quantitative VWF deficiency), accounting for 70%, 25%, and 5% of cases, respectively [3]. Type 3, characterized by a total quantitative defect of VWF and Factor VIII (FVIII), is the most severe and manifests clinically as excessive mucocutaneous and musculoskeletal bleeding including muscle hematomas, hemarthroses, and joint bleeding [4].

VWF plays a critical role in primary hemostasis by promoting adhesion and aggregation of platelets and in secondary hemostasis by acting as a transporter and stabilizer for FVIII, an essential component of the intrinsic clotting cascade. Therefore, VWF deficiency may result in primary and secondary hemostatic defects leading to bleeding disorders [5]. VWD is typically diagnosed based on the patient’s medical history and laboratory examination including screening tests (e.g., evaluation of bleeding time (BT), activated partial thromboplastin time (APTT), and platelet count (PC)), confirmatory tests (e.g., evaluation of VWF function using ristocetin cofactor assay (VWF:ristocetin cofactor (RCo)), VWF protein concentration immunoassay (VWF:antigen (Ag)), and FVIII coagulation assay (FVIII:C)); and specialized tests for characterization of VWD type (e.g., structural assays such as VWF multimer analysis and VWF propeptide (VWFpp), functional assays such as VWF binding to platelet GPIb, collagen (VWF:CB), or FVIII (VWF: FVIIIB)) [6].

Surgical interventions present a critical hemostatic challenge in patients with VWD, necessitating careful perioperative management to minimize the risk of bleeding. Although previous studies have reported minor oral surgical treatment or dental extraction in patients with VWD, there is limited evidence of orthognathic surgeries in this population [4,7]. The current case report focuses on four patients with VWD (including one with type 3 VWD) who were successfully treated using orthognathic surgical intervention.

## Case presentation

Case 1

**Figure 1:**
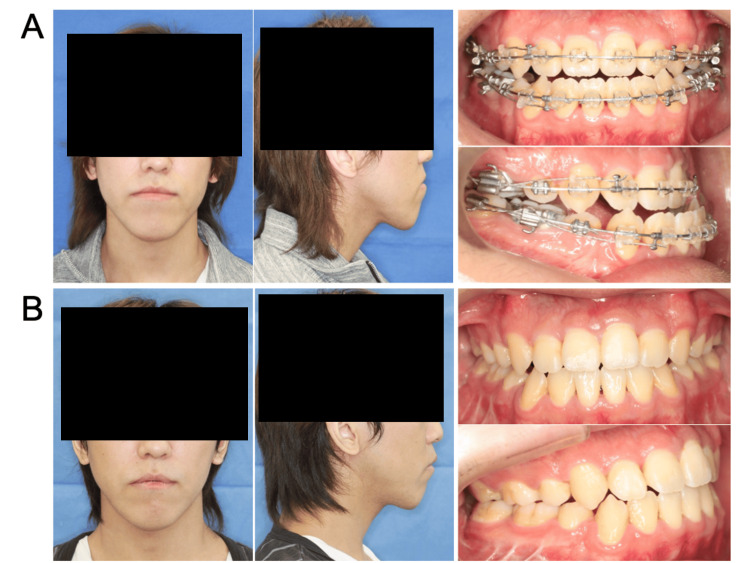
Extra- and intraoral photographs of Case 1 (A) preoperatively and (B) one year postoperatively.

A 28-year-old male patient was referred to our hospital for orthognathic surgical treatment of a skeletal class Ⅲ malocclusion in July 2013. The patient exhibited uncontrollable bleeding during presurgical orthodontic treatment that included the extraction of an impacted wisdom tooth in 2011. Following a detailed examination at the Department of Hematology at another facility, the patient was diagnosed with type 1 VWD and was transferred to our hospital where a surgical intervention plan was developed in collaboration with hematologists. Intraoral examination after presurgical orthodontic treatment showed a 1 mm deviation of the mandibular dental midline to the right, an overjet of 0 mm, and an overbite of −5 mm (Figure 1). 

Preoperative hematological examination showed that the patient’s hemoglobin (Hb) levels, white blood cell count (WBC), PC, and prothrombin time (PT) were normal, while the activated partial thromboplastin time (APTT) was prolonged at 39.3 seconds (Table 1).

**Table 1:** Patient demographics and laboratory findings at baseline (Case 1). Hb = hemoglobin; RBC = red blood cell count; WBC = white blood cell count; PT = prothrombin time; APTT = activated partial thromboplastin time; FVIII:C = factor VIII procoagulant activity

Parameter	Lab values	Reference range
Hb (g/dL)	16.0	11.0-17.0
RBC (×10^6 / uL)	5.37	3.7-5.4
WBC (×10^3 /uL)	6.6	2.7-8.8
Platelet count (×10^3 /uL)	229	140.0-340.0
PT (sec)	12.6	12.0±2.0
APTT (sec)	39.3	30.0±5.0
FVIII:C (%)	61.4	80-120
VWF ristocetin (%)	34	60-170
VWF antigen activity (%)	39	50-155

Furthermore, the baseline FVIII:C (factor VIII procoagulant activity), VWF ristocetin, and VWF antigen activity were 61.4%, 34%, and 39%, respectively. After discussing potential complications with the patient, written informed consent for surgical treatment was collected and a bilateral sagittal split ramus osteotomy (BSSO) was carried out under general anesthesia in May 2015. A FVIII/VWF concentrate (Confact F; KM Biologics, Kumamoto, Japan) infusion protocol was developed by the hematologists, and 1,000 units of Confact F was infused every 12 hours starting from the morning of the surgery up to postoperative day 6. As per the model surgery, the mandible was set back by 8.5 mm on the right side and 5.5 mm on the left side. The total surgical duration was 238 minutes, and no abnormal intraoperative bleeding was observed (total blood loss: 638 mL). The drain was removed on postoperative day 2, and the patient’s FVIII and VWF activities were seen to be 122% and 72%, respectively, on postoperative day 5. No abnormal bleeding was observed during the hospital stay, and the patient was discharged on postoperative day 7.

Case 2

**Figure 2:**
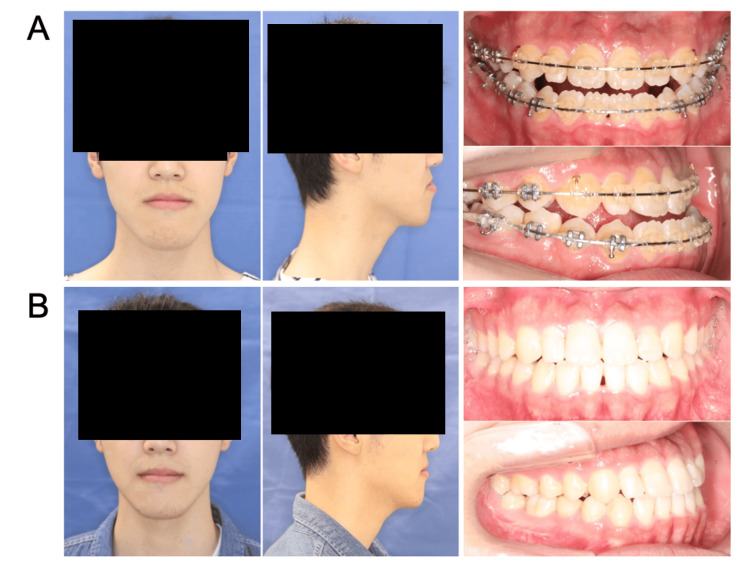
Extra- and intraoral photographs of Case 2 (A) preoperatively and (B) one year postoperatively.

A 19-year-old male patient was referred by an orthodontist for orthognathic surgical treatment of a skeletal class Ⅲ malocclusion. The patient had a history of type 3 VWD and was under the care of the hematology department at our hospital where he had previously received on-demand Confact F for epistaxis, gingival bleeding, and frequent ankle joint bleeding. Preoperative examination showed bilateral class Ⅲ malocclusion: −1 mm overbite and −2 mm overjet (Figure 2), normal hemoglobin level, WBC count, PC, and PT, and prolonged APTT (86.1).

The patient’s baseline VIII:C, VWF antigen, and VWF ristocetin activities were 0.9%, 5%, and 6%, respectively, and he was negative for FVIII inhibitors (Table 2).

**Table 2:** Patient demographics and laboratory findings at baseline (Case 2). Hb = hemoglobin; RBC = red blood cell count; WBC = white blood cell count; PT = prothrombin time; APTT = activated partial thromboplastin time; FVIII:C = factor VIII procoagulant activity

Parameter	Lab values	Reference range
Hb (g/dL)	16.4	11.0-17.0
RBC (×10^6 / uL)	5.37	3.7-5.4
WBC (×10^3 /uL)	5.0	2.7-8.8
Platelet count (×10^3 /uL)	249	140.0-340.0
PT (sec)	11.8	12.0±2.0
APTT (sec)	86.1	30.0±5.0
FVIII:C (%)	0.9	80-120
VWF ristocetin (%)	6	60-170
VWF antigen activity (%)	5	50-155

After consulting with the hematologists and anesthesiologist, the surgical details (including feasibility under strict systemic management and potential complications) were discussed with the patient, and consent for surgery was collected. Thereafter, BSSO was carried out under general anesthesia in August 2019. In accordance with the perioperative protocol developed by the hematologists, 2000 IU of Confact F was administered preoperatively, followed by 1,000 IU administered every 12 hours postoperatively until day 9. During surgery, the mandible was set back by 8.5 mm on the right side and 5.5 mm on the left side. The total surgical duration was 279 minutes and the total volume of blood lost was 834 ml. The patient was intubated for airway management and transferred to the intensive care unit (ICU). Fiberoptic laryngeal evaluation one day after surgery revealed slight laryngeal edema and, therefore, extubation was delayed to postoperative day 2 once the edema had decreased. The patient was discharged from the ICU thereafter. Epistaxis was seen to occur after extubation and this was controlled by applying direct pressure. The patient’s FVIII activity and VWF:RCo were 36.2% and 55%, respectively; therefore, an additional 1,000 IU of FVIII/VWF concentrate was administered on postoperative day 2 . The drain was removed on postoperative day 3 and the patient was able to start eating orally. His FVIII and VWF activities on postoperative day 5 were 122% and 72%, respectively, and he was ultimately discharged from the hospital on postoperative day 9.

Case 3

**Figure 3:**
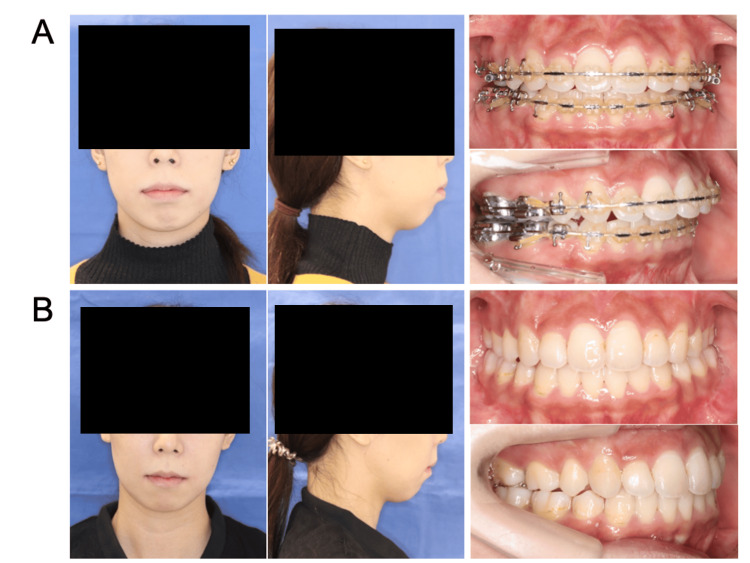
Extra- and intraoral photographs of Case 3 (A) preoperatively and (B) one year postoperatively.

A 26-year-old female patient was referred to our hospital for orthognathic surgical treatment of a skeletal class Ⅱ malocclusion in October 2014. She was scheduled to undergo a Le Fort Ⅰ osteotomy and BSSO after completion of preoperative orthodontic treatment. Presurgical screening and examination showed severe mandibular retrusion, convex facial profile, a 2 mm overbite, a 7 mm overjet (Figure 3), prolonged APTT of 36.8 seconds, and normal PT, Hb level, and PC.

Detailed hematological examination showed low FVIII:C (66.6%), VWF ristocetin (28%), and VWF antigen (67%) activity and a diagnosis of type 1 VWD was made (Table 3).

**Table 3:** Patient demographics and laboratory findings at baseline (Case 3). Hb = hemoglobin; RBC = red blood cell count; WBC = white blood cell count; PT = prothrombin time; APTT = activated partial thromboplastin time; FVIII:C = factor VIII procoagulant activity

Parameter	Lab values	Reference range
Hb (g/dL)	12.5	11.0-17.0
RBC (×10^6 / uL)	4.03	3.7-5.4
WBC (×10^3 /uL)	4.2	2.7-8.8
Platelet count (×10^3 /uL)	163	140.0-340.0
PT (sec)	11.5	12.0±2.0
APTT (sec)	36.8	30.0±5.0
FVIII:C (%)	66.6	80-120
VWF ristocetin (%)	28	60-170
VWF antigen activity (%)	67	50-155

Consent for surgery was collected after informing the patient about the precautionary measures required to manage pre- and postoperative bleeding. Approximately 800 mL of autologous blood was collected prior to surgery as blood loss was expected. In October 2019, following preoperative administration of 1,000 IU of Confact F, a multidisciplinary team that included hematologists and anesthesiologists carried out a Le Fort Ⅰ osteotomy and BSSO under general anesthesia. The total surgical duration was 375 minutes and no abnormal intraoperative bleeding was observed (total blood loss: of 554 mL). The patient was transferred to the ICU where he was intubated for airway management. Extubation was carried out on postoperative day 2 after confirmation of no laryngeal edema using fiberoptic laryngoscopy, and the patient was discharged from the ICU thereafter. Confact F transfusion (1,000 units/24 hours) was continued from postoperative days 1-5 and the drain was removed on day 3 to allow the patient to start eating orally. The patient’s FVIII and VWF activities on day 5 were 83.3% and 99%, respectively and her postoperative recovery was uneventful with no hemorrhagic complications. The patient was discharged from the hospital on day 9.

Case 4

**Figure 4:**
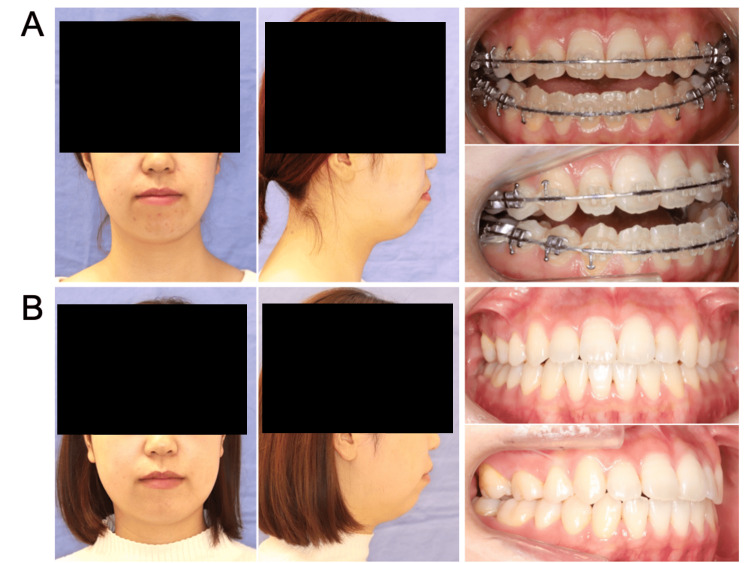
Extra- and intraoral photographs of Case 4 (A) preoperatively and (B) one year postoperatively.

A 29-year-old female patient with no significant medical history was referred to our hospital for orthognathic surgical treatment of a skeletal class Ⅲ malocclusion in July 2019. The patient was scheduled to undergo preoperative orthodontic treatment followed by BSSO under general anesthesia. Presurgical examination and hematological screening showed mandibular prognathism, concave facial profile, bilateral angle class III malocclusion, a −3 mm overbite, a −3 mm overjet (Figure 4), prolonged APTT (35.5 seconds), and low FVIII:C (53.1%), VWF ristocetin (20%), and VWF antigen (35%) activity (Table 4).

**Table 4:** Patient demographics and laboratory findings at baseline (Case 4). Hb = hemoglobin; RBC = red blood cell count; WBC = white blood cell count; PT = prothrombin time; APTT = activated partial thromboplastin time; FVIII:C = factor VIII procoagulant activity

Parameter	Lab values	Reference range
Hb (g/dL)	13.8	11.0-17.0
RBC (×10^6 / uL)	4.59	3.7-5.4
WBC (×10^3 /uL)	5.0	2.7-8.8
Platelet count (×10^3 /uL)	247	140.0-340.0
PT (sec)	13.4	12.0±2.0
APTT (sec)	35.5	30.0±5.0
FVIII (%)	53.1	80-120
VWF ristocetin (%)	20	60-170
VWF antigen activity (%)	35	50-155

Based on these findings, the patient was diagnosed with type 1 VWD. Consent for surgery was collected from the patient after discussing potential perioperative complications, and BSSO was performed in February 2022. In accordance with the infusion protocol developed by the hematologists, 1,000 IU of Confact F was administered presurgery. The total surgical duration was 216 minutes, and no abnormal intraoperative bleeding was observed (total blood loss: 154 mL). Confact F administration (dose: 1,000 IU) was carried out every 24 hours on postoperative days 2 and 3, and the drain was removed on postoperative day 2 (Table 5). The patient’s VIII and VWF activities on day 5 were 132% and 145%, respectively, and her perioperative course was uneventful with no hemorrhagic complications. The patient was discharged from the hospital 6 days after surgery.

**Table 5:** Summary of treatment of the four patients. L1 = Le fort 1 osteotomy; BSSO = bilateral sagittal split ramus osteotomy; VWD = Von Willebrand’s disease

Case	Age	Sex	VWD type	Technique	Surgical duration (minutes)	Blood loss (ml)	Management
Case 1	28	M	1	BSSO	238	638	Confact F
Case 2	19	M	3	BSSO	279	834	Confact F
Case 3	26	F	1	L1; BSSO	375	554	Confact F; autologous blood transfusion
Case 4	29	F	1	BSSO	216	154	Confact F

## Discussion

VWD is typically diagnosed based on observation of clinical and biological findings. However, some patients with VWD may not exhibit symptoms of bleeding disorder, suggesting the potential presence of undiagnosed patients in the general population. In the current report, three out of four patients were diagnosed with VWD during preoperative screening for orthognathic surgery.

VWD treatment primarily aims to correct dual hemostatic defects caused by abnormalities or deficiencies in VWF and FVIII by increasing their activity using autologous replacement therapy with desmopressin (DDAVP) and allogenic replacement therapy with FVIII/VWF concentrates. DDAVP is an antidiuretic hormone derivative that can be used to boost plasma FVIII and VWF levels; however, its efficacy in increasing VWF activity is often mild, delayed in onset, and lasts for a short duration [8]. Moreover, repeated administration of DDAVP can lead to a decrease in therapeutic efficacy, suggesting that it may be unsuitable for use during major surgical interventions (e.g., orthognathic surgery) [9]. Previous evidence suggests that it is also associated with an increased risk of onset or transient aggravation of thrombocytopenia in type 2B patients and a lack of efficacy in type 3 and type 2N patients [10]. FVIII/VWF concentrates are effective in patients with severe bleeding disorders, those undergoing major surgical interventions, as well as type 2B, 2N, and 3 patients where DDAVP is contraindicated or ineffective. However, 5-10% of type 3 patients exhibit the presence of VWF inhibitors that render treatment ineffective and often result in anaphylactic reactions upon subsequent exposure [11]. Therefore, the establishment of individual patient response to FVIII/VWF concentrate using test infusions prior to treatment is essential.

In the current study, the response pattern of Case 2 was already known as he had been previously tested for anti-VWF antibodies and had also undergone FVIII/VWF concentrate treatment multiple times during his childhood. Pharmacokinetic assessment of response to FVIII/VWF concentrates was carried out in all other patients prior to treatment. The Japanese treatment guidelines for VWD recommend using an initial dose of 50-60 IU VWF:RCo/kg (target peak level: 100% VWF:RCo)) of FVIII/VWF concentrates and a maintenance dose of 20-40 IU VWF:RCo/kg administered every 8-24 hours for 7-14 days in patients undergoing major surgery [12]. Furthermore, VWF and FVIII activity should be maintained at ≥50% (0.5 IU/mL) from postoperative day 2. In the current study, FVIII/VWF concentrate containing 2,500 units of VWF:RCo in 1,000 units was administered before and after surgery to ensure maintenance of VWF and FVIII activity above 50%. This method was effective until postoperative day 4 in all patients except Case 2 who exhibited VWF:RCo activity >55% and FVIII activity <50% (i.e., 36.2%) on postoperative day 1 and epistaxis after extubation on postoperative day 2, necessitating additional infusion of FVIII/VWF concentrate. This suggests that the dosage and timing of FVIII/VWF concentrate administration varies with the severity of VWD and the surgical treatment being carried out.

The incidence rates of perioperative complications associated with orthognathic surgery range between 6.1% and 9.0% [13,14]. In particular, severe bleeding (i.e., >1,000 g) as a consequence of injury to major arteries (e.g., palatal or inferior alveolar arteries) has been reported to occur in 0.085%-1.1% of patients, necessitating immediate application of pressure packing, use of topical hemostatic agents, and ligation of the affected blood vessel [13,14].

Surgical complications in patients with VWD include prolonged and possibly life-threatening blood loss, highlighting the importance of a detailed assessment of the risk of bleeding and the development of management strategies for unexpected intraoperative and postoperative hemorrhage. The literature review identified eight patients with bleeding disorders (i.e., three with VWD and five with hemophilia A and factor XI deficiency) who had previously undergone orthognathic surgery (Table 6) successfully with no perioperative hemorrhagic complications.

**Table 6:** Review of literature on orthognathic surgeries conducted in patients with bleeding disorders. L3 = Le fort 3 osteotomy; L1 = Le fort 1 osteotomy; BSSO = bilateral sagittal split ramus osteotomy; FFP = fresh frozen plasma; EACA = epsilon-aminocaproic acid; VWD = Von Willebrand’s disease

Author (year)	Comorbidity	Age (year)	Sex	Technique	Surgical duration (minutes)	Blood loss	Management
Ilankovan et al., 1990 [15]	Factor XI deficiency;	23	M	L3	Not mentioned	Not mentioned	FFP
	Mild VWD	23	F	Bilateral body ostectomy	Not mentioned	Not mentioned	Factor VIII replacement
	Mild VWD	18	F	Three-part L1; BSSO; genioplasty	Not mentioned	Not mentioned	Factor VIII replacement
Todd et al., 1993 [16]	Factor XI deficiency	16	F	L1; BSSO	Not mentioned	400 ml	FFP; EACA
Abramowicz et al., 2008 [17]	Hemophilia A	16	F	BSSO; genioplasty; wisdom teeth extraction	Not mentioned	400 ml	Recombinant factor VIII replacement; aminocaproic acid
Lee et al., 2015 [18]	Factor XI deficiency	25	F	L1; BSSO	250	560 ml	FFP; autologous blood transfusion; tranexamic acid
Kasahara et al., 2022 [7]	Mild hemophilia A	27	M	BSSO	140	150 ml	Recombinant factor VIII replacement
	VWD	34	F	BSSO	137	Small amount	Not mentioned

Three of these patients received adjunctive treatment with tranexamic acid and epsilon-aminocaproic acid [15-17]. The clinical relevance of tranexamic acid-associated decrease in surgical bleeding remains debatable. Previous meta-analyses of RCTs showed that intravenous or topical administration of tranexamic acid can effectively reduce perioperative bleeding and transfusion rates in patients undergoing orthognathic surgery [19]. In contrast, a recent study showed that although tranexamic acid effectively controlled intraoperative blood loss and improved the quality of the surgical site, it did not exhibit any effect on postoperative levels of hemoglobin or hematocrit or the need for blood transfusion [20]. Appropriate management of patients with bleeding disorders includes the presurgical development of management strategies aimed at minimizing bleeding by shortening the surgical duration and using local hemostatic agents [7,16]. In the current study, intravenous or topical administration of tranexamic acid was carried out postsurgically in all patients, and local hemostatic agents were used during surgery to control bleeding.

Many young adults do not experience general anesthesia and, therefore, may not have undergone blood tests at a hospital, highlighting the importance of preoperative screening. The safety of surgical interventions has also improved considerably due to rapid developments in the surgical techniques, general anesthetics, and devices used. Nevertheless, as orthognathic surgery is an elective procedure, individualized treatment protocols should be developed following adequate consultation with the clinician and patient regarding their medical history to determine whether the surgery is necessary.

The findings of the current study emphasize the importance of a multidisciplinary approach to orthognathic surgery in patients with bleeding disorders such as VWD. Close communication between anesthetists, surgeons, and hematologists is essential to facilitate the development of individualized plans for the perioperative management of these patients.

## Conclusions

The current report focuses on four patients with VWD who underwent orthognathic surgery successfully, with a multidisciplinary team including hematologists and anesthetists ensuring appropriate perioperative management of hemostasis. The findings of this study also highlight the importance of preoperative screening.
